# Spawning behavior of Arctic charr (*Salvelinus alpinus*): Spawning synchrony, vibrational communication, and mate guarding

**DOI:** 10.1002/ece3.4277

**Published:** 2018-07-20

**Authors:** Magnus B. Brattli, Torvald B. Egeland, Jarle T. Nordeide, Ivar Folstad

**Affiliations:** ^1^ Department of Arctic and Marine Biology UiT – The Arctic University Tromsø Norway; ^2^ Faculty of Biosciences and Aquaculture Nord University Bodø Norway

**Keywords:** female choice, mate guarding, reproductive behavior, spawning synchrony, sperm competition, vibrational communication

## Abstract

A mismatch in synchrony between male and female gamete release in external fertilizers can result in reduced or failed fertilization, sperm competition, and reduced paternity. In Arctic charr (*Salvelinus alpinus*), males can adopt either a guard or sneak tactic resulting in both pre‐ and postcopulatory competition between males with alternative reproduction tactics. Here, spawning behavior of free‐living Arctic charr was video‐recorded, and their reproductive behavior was analyzed. From evaluating 157 spawning events, we observed that females mainly spawned with a guarding male and that the female and the guarding male synchronized timing of gamete release under sperm competition. Although sneakers spawned with higher synchrony than the guarding male in single‐male spawning events, the average sneaker released his milt less synchronized with the female than the guarding male under sperm competition. Approximately 50% of the recorded spawning events occurred under sperm competition, where each event included an average of 2.7 males. Additionally, sneakers were more exposed to sperm competition than guarding males. An influx of males, in close proximity to the female, occurred during the behavioral sequences leading up to egg release, but this influx seemed not dependent on egg release, suggesting that something else than gonadal product attracts sneaker males to the spawning female. Just before and during the actual release of gametes, the spawning couple vibrates their bodies in close contact and it seems likely that this vibrational communication between the spawning couple, which results in a larger amplitude sound wave than seen under regular courting, reveals time of gamete release to sneaker males. Thus, vibrational communication may enable synchrony between the guarding male and the female, and this might be traded against the cost of higher detectability from surrounding sneaker males, eavesdropping in close proximity.

## 1 INTRODUCTION

In a blink of an eye, hundreds of eggs and millions of sperm are released in open water when external fertilizers spawn. In salmonids, the micropyle stays open for approximately 40 s before osmotic swelling blocks the entrance and prevents sperm from fertilizing the egg (Billard, 1992; Ginsburg, 1963; Hoysak & Liley, 2001) and the first sperm cell to reach the egg and enter the micropyle fertilizes the egg (Hoysak & Liley, 2001; Kobayashi & Yamamoto, 1981; Yanagimachi, Cherr, Pillai, & Baldwin, 1992). Given the abovementioned constraints, a mismatch between male and female gamete release can result in reduced or failed fertilization. Additionally, given sperm competition, the blocking of the micropyle by sperm from one male might result in reduced paternity for other males (Kobayashi & Yamamoto, 1981). Synchrony in gamete release is therefore particularly important for external fertilizing species with eggs equipped with micropyles (Mjølnerød, Fleming, Refseth, & Hindar, 1998; Yeates, Searle, Ward, & Gage, 2007).

Annually, breeding Arctic charr (*Salvelinus alpinus*) gather on specific spawning grounds to reproduce by shedding their gonadal products into the external environment. Here, on shallow waters, females ready to release their eggs seem to attract males to their desired spawning site. The spawning males often adopt different size‐dependent mating tactics, either dominant (guarding) or subordinate (sneaker) (Figenschou, Rudolfsen, & Folstad, 2007; Sigurjónsdóttir & Gunnarsson, 1989; Sørum, Figenschou, Rudolfsen, & Folstad, 2011; Supporting information video S1), and their differing tactic is easily distinguished by recognizable behavioral traits (Sigurjónsdóttir & Gunnarsson, 1989). Bigger dominant males often acquire a guarding tactic, protecting and defending the spawning female against other surrounding males by aggressive traits like biting and chasing (Sigurjónsdóttir & Gunnarsson, 1989). In the presence of a guarding male, smaller subordinate males often adopt a sneaking spawning behavior circulating the spawning female and occasionally trying to court the female. The sneakers may also try to fertilize the eggs by rushing into the spawning site and releasing their milt shortly after the guarding male and the female have spawned (Sigurjónsdóttir & Gunnarsson, 1989). The males’ spawning tactics seem to be highly plastic as they can shift between guarding and sneaker behavior depending on interacting males (Liljedal & Folstad, 2003; Rudolfsen, Figenschou, Folstad, Tveiten, & Figenschou, 2006).

Conflicts between males trying to fertilize the eggs are common (Sørum et al., 2011; own unpublished data). Bigger guarding males have the advantage of spawning close to and in synchrony with the spawning female. Smaller sneaker males, on the other hand, are forced by the aggressive bigger male to spawn out of synchrony and further away from the released gonadal products of the female (Sørum et al., 2011). Guarding and synchronized spawning by the dominant male may thus leave fewer unfertilized eggs available for the sneaker males, and the eggs will also be more dispersed and difficult to fertilize. Yet, sperm competition occurs when sneaker males try to fertilize a limited number of dispersed, unfertilized eggs (Birkhead & Møller, 1998; Egeland, Rudolfsen, Nordeide, & Folstad, 2016; Sørum et al., 2011).

In species where the males show alternative reproductive tactics, reproductive behavior is of particular interest (Hoysak & Liley, 2001; Taborsky, 1998). These different behaviors are tailored to increase a male's chance to fertilize the eggs, and physiological adaptations to each tactic would involve adjustments of reproductive organs, spermatozoa, and other seminal products (Parker, 1984; Taborsky, 1998). Increasing the chance of fertilization by expressing one trait may also reduce the investment in alternative traits; therefore, a trade‐off between different traits might be expected (Taborsky, 1998). For spawning Arctic charr, sneaker males are disfavored, compared to dominant males, because of their “delayed gamete release” and increased distance to the already dispersed eggs. Yet, sneakers seem to compensate for these disadvantages by producing more sperm and sperm that also swim faster in water than the sperm from guarding males (Rudolfsen et al., 2006). However, sperm from sneakers swim slower in the water‐diluted ovarian fluid surrounding the eggs, compared to sperm from guarding males, suggesting that sperm cells of guarding males are tailored to swim in a different environment than sperm from sneakers (Egeland et al., 2016). Thus, sperm competition in charr seems to be a “loaded raffle” (Parker, 1990).

An additional advantage under sperm competition could be gained by improving synchrony in gamete release. However, high synchrony in gamete release relies on good communication between the female and the male. Many species of fish are reported to use vibrational signals to synchronize spawning (Satou, Shiraishi, Matsushima, & Okumoto, 1991). For the landlocked red salmon (*Oncorhynchus nerka*), the vibrational signals, made by trunk muscle activity during courtship between male and female, are detected and processed by the lateral line system to elicit the synchronized spawning behavior (Satou, Takeuchi, Nishii, et al., 1994). These vibrations act as timing cues enabling synchrony of the gamete release. As shown by Sørum et al. (2011), guarding and sneaker males of Arctic charr may differ in how synchronous they manage to ejaculate with the spawning female, in situations both with and without sperm competition. That is, the average time delay in gamete release under sperm competition between the guarding male, spawning in synchrony with the female, and the first sneaker was shown to be 0.68 s (Sørum et al., 2011). Females also initiated spawning with guarding males in 73.3% of all observed events, and 55.6% of the spawning events occurred under sperm competition. Yet, in Sørum et al. (2011) study, only 45 spawning events were included, and in order to increase the knowledge about spawning behavior among free‐living charr, more data are needed to be able to conduct experiments that closely mimic the actual spawning situation (see Egeland, Rudolfsen, Nordeide, & Folstad, 2015 for a first attempt).

The primary aim of this study was to collect additional and higher quality data on spawning behavior of Arctic charr. Although replicating previous observations is in itself relevant (Ioannidis, 2005; Van Bavel, Mende‐Siedlecki, Brady, & Reinero, 2016), the last decade's technological development of cameras and video quality additionally enables us to operate more cameras and hence record more spawning events at video resolutions revealing behaviors previously not documented in our population (e.g., egg eating including filial cannibalism). Moreover, the vibrations of charr during courtship and spawning leave a recordable sound track in the water column (discovered here by MBB). Recording of the sound produced during courtship and spawning—sound that previously have been searched for but not found and described in this species (Bolgan et al., 2017)—enabled an evaluation of the importance of vibrational communication for spawning synchrony and intensity of sperm competition. That is, by comparing behavioral sequences that resulted in gamete release with those that did not result in gamete release, we were able to make qualified evaluations of important attractors (gonadal products or sound) for sneaker males.

## 2 MATERIAL AND METHODS

Some of the data presented in this study have previously been analyzed and described in Sørum et al. (2011) study. In this former study, conducted in 2006–2007, spawning behavior was recorded for 69 hr and 40 min. To increase the sample for this study, recording of spawning behavior was conducted for 284 hr and 28 min during the 2016 spawning season, using the same approach as Sørum et al. (2011) but with improved camera quality enabling a more detailed evaluation of charr behavior. In total, 110 hr and 42 min of the 2016 recordings were analyzed and are presented in this study. Here, 112 new spawning events were analyzed, and the data from 2006 to 2007 and 2016 were pooled. This summed up to 180 hr and 22 min of analyzed videos resulting in 157 spawning events.

The quivering from the courtship behavior of a spawning couple made a distinguishable sound which was recorded by the recording camera. 32% of the videos from 2016 were analyzed using the sound files only to identify spawning. This resulted in the identification of 33 spawning events. The remaining 68% were analyzed by watching the video, resulting in the identification of 79 additional spawning events. To control the accuracy of using sound files only to identify a spawning, we matched the spawning events, first identified from watching the videos, with those identified (by a different person) from the sound file only. The match between the two separate methods to identify spawning events was 100% (*n* = 33), and there were no spawning events that did not have vibrational cues (*n* = 47).

### 2.1 Study site and video recordings

The study was carried out during the spawning period from mid‐September to early October in Lake Fjellfrøsvatnet, Troms, Norway (69°08′N 19°34′E). Video monitoring of spawning Arctic charr on their Lek sites was conducted at known locations in and around spawning site 3 (see Figenschou, Folstad, & Liljedal, 2004). All the eight cameras used in the survey varied in technical specifications, but all were “action sport cameras” equipped with watertight housing and a wide‐angle lens. All cameras belonged to the GoPro brand including models GoPro Hero 3 and 4 (types plus, silver, and black). Chosen setting for video quality was 1080p with 60 frames per second. The cameras recorded both image and sound, and there were only minor technical differences in camera design and housing.

When arriving at the spawning grounds, the first 5–10 min were spent studying the charr in order to identify stationary females. Once identified, cameras mounted on tripods were deployed aiming toward the stationary females that appeared to be preparing to spawn. The distance from the camera to the spawning female was approximately 0.3 to 1 meter. Recording lasted as long as the battery capacity allowed (from about 90 to 270 min), and the capacity of the memory card was only rarely a limiting factor. The recording cameras were left undisturbed on the spawning site for minimal human interference until they were replaced by new cameras. The procedure often resulted in an exchange of cameras in the early morning, before midday and in the afternoon. All recordings had to be carried out under daylight conditions, yet night and sunset hours might be the periods with the most spawning activity (Bolgan et al., 2017). Recorded videos were immediately copied to hard drives and the batteries recharged.

The spawning events took place in shallow waters (0.2–2 meters deep), often near land or on a spawning site about 100 m from land. The preferred spawning habitats consisted of small‐ to intermediate‐sized rocks covered in algae. Females ready to release their gametes hover a few centimeters above their chosen spawning site while being guarded by a dominant male. Females seem to get more stationary the closer they are to spawning, and this increases the chance of recording the actual spawning event.

### 2.2 Spawning located by sound waves

The high‐amplitude quivering of the courtship behavior of a female and a male Arctic charr could be recorded and identified as a distinct sound curve (Figures 2, 3, 4), and this sound wave was easily distinguishable from other sounds in the videos. By placing a camera close to the spawning female, the camera—closed within the watertight housing—recorded vibration as sound from spawning individuals as far as 5 to 6 meters away. As the recording camera occasionally registered sound waves from spawning individuals located in a blind angle of the camera, video was used to verify the observed sound wave and used to locate spawning events. Using the WavePad Audio Editing Software (version 6.59) to visualize and analyze the extracted sound files from a recorded spawning video, it was possible to pinpoint the exact time of a spawning. Compared to watching videos in search for spawning events, observing the sound tracks reduces the time used to discover spawning events from the videos.

**Figure 1:**
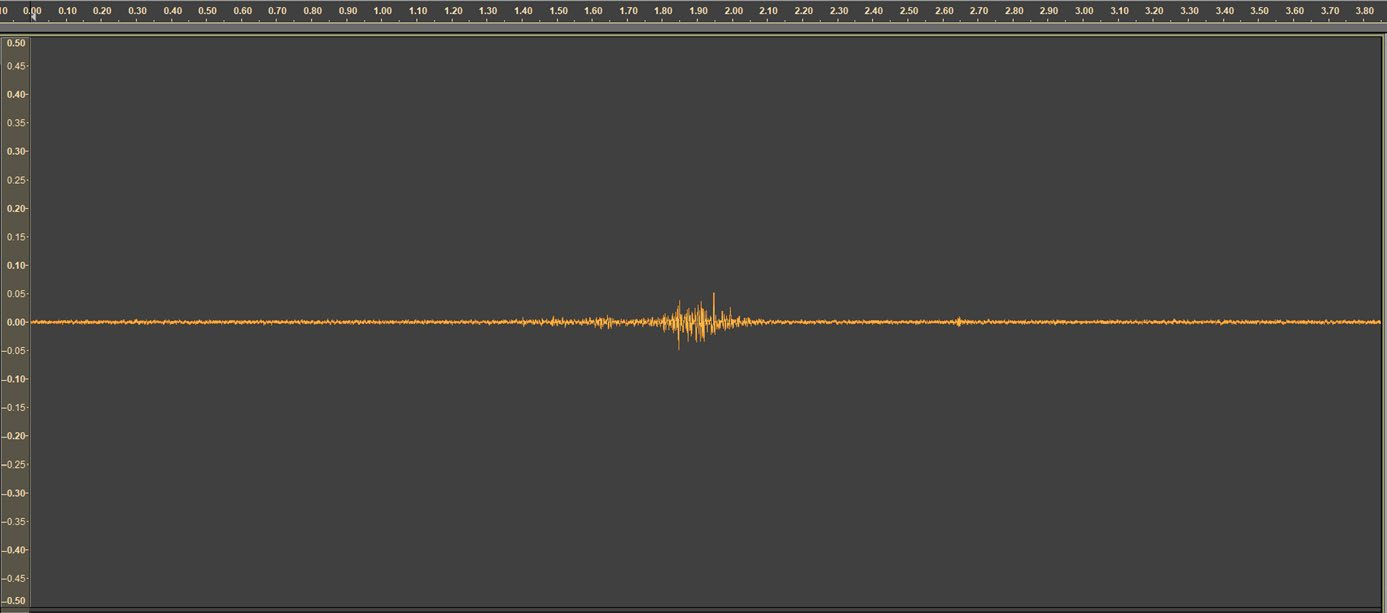
Oscillogram recorded during a courtship event (x‐axes: time in ms, y‐axes: linear scale amplitude)

**Figure 2:**
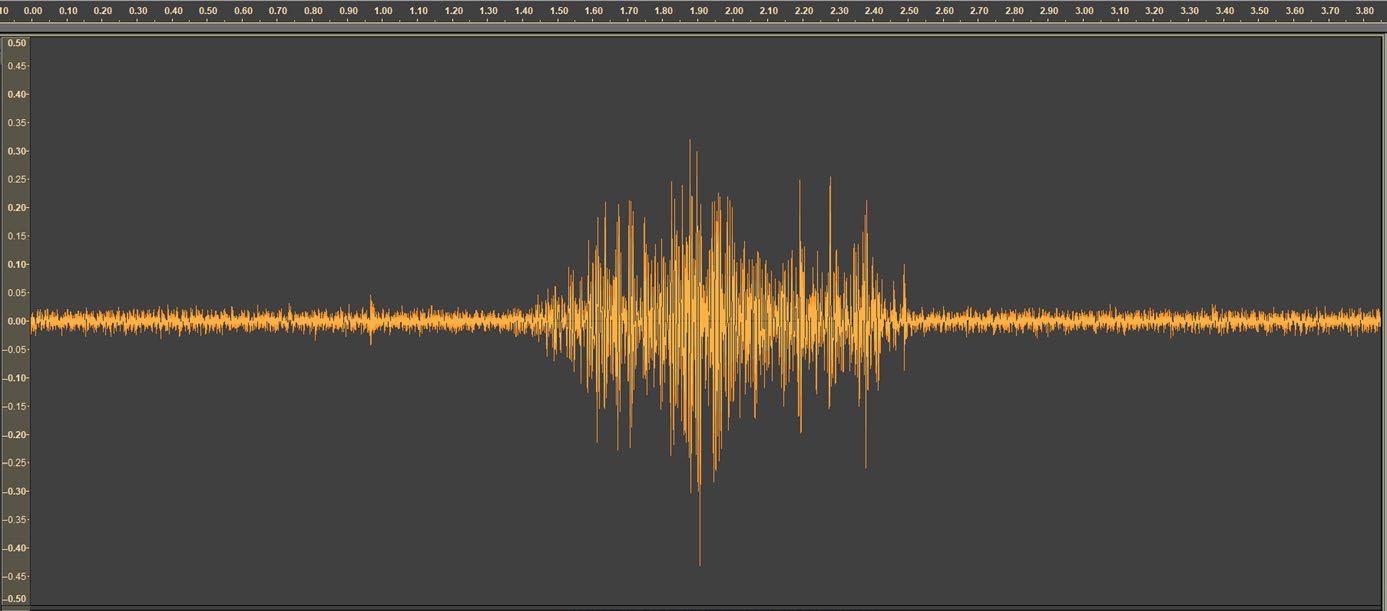
Oscillogram recorded during a single spawning event (x‐axes: time in ms, y‐axes: linear scale amplitude)

**Figure 3:**
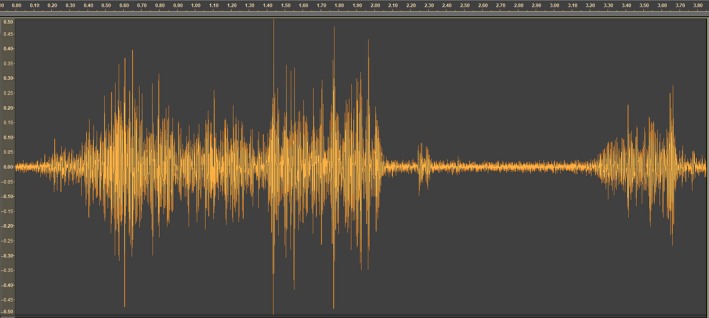
Oscillogram recorded during a spawning event with sperm competition (x‐axes: time in ms, y‐axes: linear scale amplitude)

**Figure 4:**
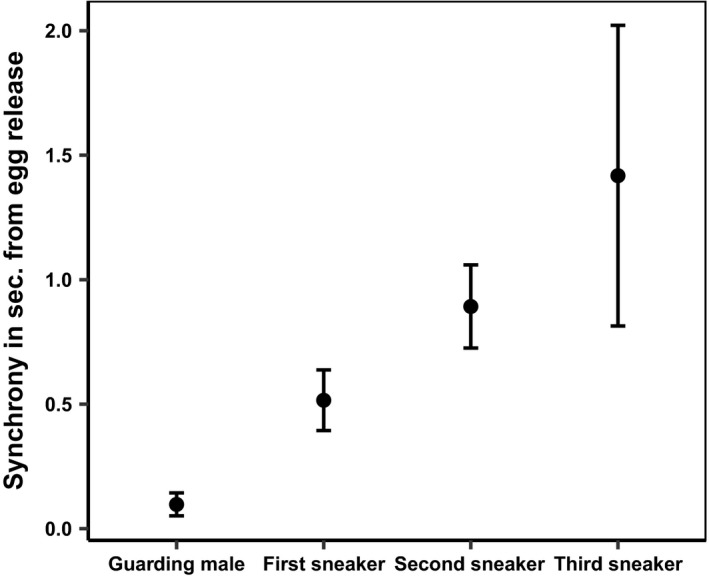
Time delay (mean ± 95% CI) from time of egg release (0) to time of milt release under sperm competition (*N* = 85, but sample size differs among male spawning tactics)

### 2.3 The spawning event and its definitions

In accordance with Sørum et al. (2011), a spawning event is defined when the following four different types of spawning behavior (adapted from Fabricius & Gustafson, 1954; Fabricius, 1953; Sigurjónsdóttir & Gunnarsson, 1989; Satou, Shiraishi, Matsushima & Okumoto, 1991; Fleming, 1996) take place (Supporting information video S2):

1. The female lies stationary close to the bottom substrate with an erected anal fin and with the upper body slightly pointing upward.
2. The male (both guarding male and sneaker) courts the female as he approaches the female from behind, and in the moment his head touches the female's tail, he initiates quivering. The males’ quivering increases as he glides forward close up to the female's body. The female often responds by quivering shortly after the quivering males touch her body.
3. Quivering increases in frequency until both the male and the female gape. The female often gapes first. Gamete release occurs at maximal mouth opening. Males’ milt can be visible as a cloud in the water, and eggs can be seen both soaring in the water and lying on the bottom substrate. Male and female propel slightly upward and forward with an open mouth and a lifted head.
4. The male and the female separate and quickly return to the spawning spot, where they start to chase away other fish from the spawning location.

### 2.4 Guarding and sneaking tactics

Stationary females tend to be more aggressive against smaller sneaker males than against bigger males employing the guarding tactic (Bolgan, O'Brien, Picciulin, Manning, & Gammell, 2016). Additionally, the guarding male is recognized by a bigger body size, a lighter dorsal color, and behavioral traits such as lying above the female, swimming slowly nearby the female, or attacking other males (Sigurjónsdóttir & Gunnarsson, 1989). The sneaker, on the other hand, is typically characterized by his smaller body size and by approaching and swimming slowly near the female (Sigurjónsdóttir & Gunnarsson, 1989). Identifying the type of mating tactic of a male in proximity to the female in a prespawning behavior is therefore easy. In the 157 recorded spawning events, every female was protected by one dominant male guarding her from the surrounding sneaker males. In cases of reproductive competition, the sneaker would either court the female to spawn without sperm competition or dart into the spawning site and release his milt in sperm competition with the guarding male.

### 2.5 Spawning synchrony

The Avidemux 2.6 video processing program (version 2.6.18) enabled the analysis of spawning synchrony and time of maximal mouth opening‐defined gamete release. Not all the spawning females were appropriately recorded, and in 16 of the total 157 recorded spawning events, the females spawned with her head pointed away from the camera or other individuals masked the gaping fish, impeding the exact measurements needed. These spawning events were excluded when estimating spawning synchrony.

### 2.6 Male density, quivering, sperm competition, and gamete release

In accordance with Sørum et al. (2011), male density was defined as the number of surrounding males within a radius of a fish length distance (approximately 25 cm) from the spawning female. The density was recorded at specific points in time from five‐seconds before to five‐seconds after female gamete release. Sperm competition was defined to occur when more than one male released milt at the same spawning event. Asynchrony in gamete release was estimated by noting time of milt release relative to time of egg release at a precision of 16.6 ms (60 frames per second). Quivering length of the courting male was estimated by noting start and stop time of the quivering. Quivering length was measured in 71 events with 17 different males.

### 2.7 “Near” spawning: Male density and vibrational communication

Examination of the videos revealed some events where the female and male(s) did not release any gametes, despite demonstrating all prespawning behaviors. Such events are hereafter termed “near” (vs. “real”) spawning events. In order to compare male behavior leading up to “real” and “near” spawning events, a total of 20 near spawning events were analyzed. The events were chosen to fulfill the spawning criteria, and when a female had multiple “near” spawning events, we chose one of the events by random. The density of neighboring males at “near” spawning events was examined in a similar way as in “real” spawning events (see above). Egg release, which did not happen in “near” spawning events, was estimated to “occur” after a quivering period comparable to that recorded from actual spawning events. That is, we used average length of the quivering period leading up to “real” spawning to estimate the likely spawning time at the near spawning events.

### 2.8 Statistical analysis

All statistical analyses were performed using R v. 3.4.2 (R Core Team, 2015). Binomial tests (to compare two proportions) were used to examine whether females spawned equally often with guarding and sneaker males. As we were not able to fit a generalized linear mixed model (GLMM) when including all spawning events, spawning synchrony between females and males (i.e., whether males or females released their gametes first) was tested with one‐sample t‐test. Spawning synchrony in sperm competition and single spawning events was examined by generalized linear mixed models (GLMM) using the lmer function in the lme4 package in R (Bates, Bolker, & Walker, 2014). In these models, time since female egg release was used as a response variable, male status as a fixed factor, and female id as a random factor. Risk (i.e., probability of experiencing sperm competition) and intensity (i.e., number of competing males) of sperm competition were tested using binomial tests. GLMM with the glmer function in the lme4 package (Bates et al., 2014) was used to analyze the male density around the spawning female. Here, we used a Poisson's distribution with the number of males as a response variable, time and spawning type as fixed factors, and female id as a random factor. Finally, Spearman's rank test was used to examine the potential correlations between the length of the quivering period and (a) the number of males releasing milt, (b) density of males around female, and (c) the relative increase in the number of males in the vibrational time span.

We recorded multiple spawning events of several of the females, and in order to reduce problems with pseudoreplication (Colegrave & Ruxton, 2017; Hurlbert, 1984) in the binomial and Spearman's rank tests, we used the average values from the observations of each individual female. In the t‐tests, we corrected the degrees of freedom according to the number of females we had recorded spawning events from instead of the number of actual spawning events recorded. In the GLMMs, pseudoreplication is not a problem as female id was included as a random factor. We checked the model fit using the visual examination of normal probability plots and residual plots.

## 3 RESULTS

### 3.1 Courtship

The numbers of female spawning events occurring when courted by a guarding male, when courted by a sneaker male, or when courted by both simultaneously were 124 (78.9%), 30 (19.1%), and 3 (1.9%), respectively. The percentage of spawning events with sperm competition was 53.5% (*n* = 157). The female spawned more often when courted by guarding males than by sneaker males, both under sperm competition and under single spawning events (binomial test comparing two proportions, *n* = 32, x^2^ = 92.3, *p* < 0.0001 and *n* = 29, x^2^ = 34.0, *p* < 0.0001, respectively).

### 3.2 Gamete synchrony, sperm competition, and different male tactics

The guarding male ejaculated on average 0.13 s (*SD* ± 0.18, *n* = 97) after and significantly later than the spawning female (one‐sample *t* test, t_26_ = 7.2, *p* < 0.001). The first sneaker, on the other hand, ejaculated on average 0.41 s (*SD* ± 0.47, *n* = 75) after the spawning female (one‐sample *t* test, t_20_ = 7.6, *p* < 0.001). By pooling all the values of spawning sneakers, the average sneaker was also observed to spawn significantly later than the female (one‐sample *t* test, t_20_ = 10.8, *p* < 0.001), with a delay of 0.6 s (*n* = 106).

**Figure 5:**
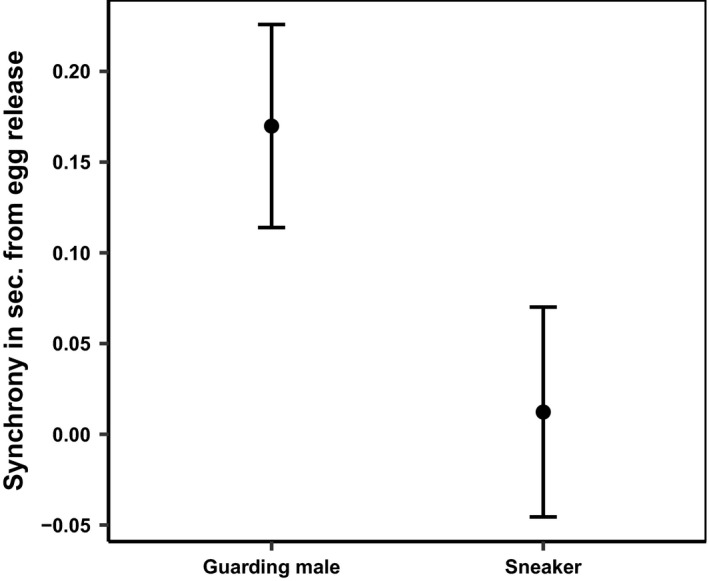
Time delay (mean ± 95% CI) for guarding (*n* = 41) and sneaker (*n* = 15) male milt release in single‐male spawning events, relative to female egg release (0)

The guarding male released milt before the sneaker males in 73 (89.1%) of the 85 analyzed spawning events with sperm competition. The difference in timing of milt release between the guarding male and the first, second, and third sneakers was significant (Figure 4, Table 1). Yet, in single spawning events, sneaker milt was released more in synchrony with the female egg release than milt released by the guarding males in single spawning events (Figure 5, Table 2). In 72.8% of the spawning events, the female was the first to release gametes.

**Table 1:** Results from a linear mixed‐effects model for spawning synchrony between the female and guarding male and first sneaker; second sneaker; and third sneaker in spawning events with sperm competition

Response	Predictor	Estimate	St. error	95% CI	*p*
Time since female egg release	Intercept	0.07	0.06	−0.04 to 0.19	0.21
	First Sneaker	0.45	0.07	0.31 to 0.58	<0.0001
	Second sneaker	0.82	0.09	0.65 to 1.00	<0.0001
	Third sneaker	1.31	0.15	1.03 to 1.60	<0.0001

*Note*

Fixed effects are presented with estimate parameters including standard error (St. error), 95% confidence intervals (95% CI), and *p*‐values (*p*) (*n* = 146).

**Table 2:** Results from a linear mixed‐effects model comparing spawning synchrony of the guarding males versus sneaker males in solitary spawning situations (i.e., without sperm competition)

Response	Predictor	Estimate	St. error	95% CI	*p*
Time since female egg release	Intercept	0.17	0.02	0.12 to 0.21	<0.0001
	Sneaker	−0.16	0.05	−0.26 to −0.06	<0.0001

*Note*

Fixed effects are presented with estimate parameters including standard error (St. error), 95% confidence intervals (95% CI), and *p*‐values (*p*) (*n* = 56).

### 3.3 Intensity and risk of sperm competition

Sperm competition can be expressed as risk (probability of experiencing sperm competition) or intensity (the number of competing males) of sperm competition. The risk of sperm competition was 75.9% (230 of 303 ejaculates experienced sperm competition). Thus, more ejaculates were released in sperm competition than in single spawning events (binomial test to compare two proportions, x^2^ = 160.63, *p* < 0.0001). The average intensity of sperm competition was 2.69 (range 2–6). When including the single spawning events, the average numbers of males releasing milt decreased to 1.93 (range 1–6).

### 3.4 Male density when females spawn

**Figure 6:**
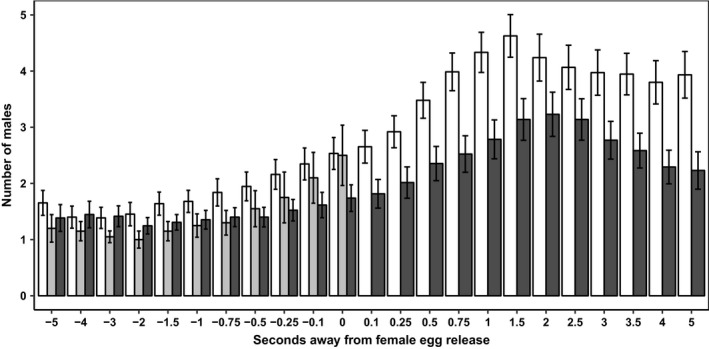
Number (mean ± 95% CI) of males in proximity to the spawning female in spawning events with sperm competition (white bars, *n* = 84), in near spawning events (gray bars, *n* = 20), and in single spawning events (black bars, *n* = 73). Zero seconds indicates time of female egg release

The male density in proximity to the spawning female started to increase a few seconds before the gamete release (Figure 6). In spawning events with sperm competition, the density of males reached its maximum 1.5 s after egg release (mean = 4.63 males per female, median = 4, range 1–9). At the time of egg release, the mean number of surrounding males was 2.64 (median = 2, range 1–7). Males released milt from 0.7 s before egg release to 2.5 s after egg release. During this time window, there was a mean increase of 2.2 males (120%) in proximity to the female. When only one male spawned, the density of males reached its maximum 2 s after egg release (mean 3.17 males per female, median = 3, range 1–9), and at the time of egg release, the mean number of males was 1.74 (median = 1, range 1–5). Overall, there were fewer surrounding males in single spawning events than in spawning events with sperm competition (*p* < 0.0001, Table 3), and the increase of males over time was also smaller in single spawning events than in spawning events with sperm competition (*p* < 0.0001, Table 3). There was no relationship between the length of the quivering period and (a) the number of males releasing milt (Spearman's rank test, S = 660.5, *p* = 0.46), (b) the number of males in proximity to the female at egg release (Spearman's rank test, S = 790.4, *p* = 0.91), or (c) the relative increase of males in the vibrational time span (Spearman's rank test, S = 645.9, *p* = 0.422).

**Table 3:** Results from a generalized linear mixed‐effects model for the number of males in close proximity to the female over time (std time) in spawning with sperm competition, “near” spawning events (near) and single spawning events (single)

Response	Predictor	Estimate	St. error	95% CI	*p*
Number of males	Intercept	0.89	0.05	0.79 to 0.99	<0.0001
	std time	0.73	0.03	0.67 to 0.79	<0.0001
	Near	−0.16	0.11	−0.34 to 0.07	0.21
	Single	−0.2	0.03	−0.25 to −0.14	<0.0001
	std time x near	0.42	0.24	−0.6 to 0.89	0.08
	std time x single	−0.18	0.05	−0.28 to −0.09	<0.0001

*Note*

Fixed effects are presented with estimate parameters including standard error (St. error), 95% confidence intervals (95% CI), and *p*‐values (*p*) (*n* = 157).

### 3.5 Male density when females do not spawn

In “near” spawning events, there was a significant increase in the density of males in the four‐seconds preceding estimated female “gamete release” (Pearson's correlation test, *r* = 0.374, *p* < 0.0001, *n* = 220, Figure 6). However, compared to “real” spawning events, “near” spawning events had on average fewer males present in the time span from 2 to 0.75 s before “female gamete release” (Figure 6). At the estimated time of “egg release,” the mean number of males in proximity to the female was similar to spawning events with egg release (mean ± *SD*, “near” spawning: 2.5 ± 1.15, “real” spawning: 2.64 ± 1.27).

### 3.6 Sound‐producing vibrational communication related to courtship and spawning

The charr produced three different sound waves under courtship and spawning; these sound waves were easy to distinguish from other sound waves in the videos. These three sound waves were when a male courts a female (Figure 1), a single spawning event (Figure 2), and a spawning event with sperm competition (Figure 3). The different sound waves are identifiable by the amplitude and the duration of the sound (Supporting information video S3).

## 4 DISCUSSION

Females spawned more frequently when courted by the guarding males than when courted by the sneaker males. Additionally, like Sørum et al. (2011), we found that the spawning female experienced a high level of synchrony in the timing of gamete release with the courting male. The females, which most often released gametes first, were shortly followed by the guarding or sneaker(s) ejaculation. The majority of ejaculates was released under sperm competition. However, ejaculates from guarding and sneaker males differed in the risk of sperm competition with a higher intensity of sperm competition among sneaker males’ ejaculates. Additionally, as density of males in proximity to the female increased right before eggs were “shed” in both “real” and “near” spawning events, there must be some form of communication involved in a “spawning” which is not related to the actual gamete release per se.

### 4.1 Female preference

In the present study, the majority of females spawned when courted by the guarding males (in 125 of 157 events), guarding males commonly being larger (Sigurjónsdóttir & Gunnarsson, 1989). Size is a well‐known mate choice criterion in salmonids (Bolgan, O'Brien, Picciulin, et al., 2016), and females have in the presence of small males been shown to delay their spawning allowing larger males to displace the small males (Blanchfield & Ridgway, 1998; Gaudemar, Bonzom, & Beall, 2000). Male size is also known to be an important factor for eliciting the behavior leading to spawning. In a study of Atlantic salmon (*Salmo salar*), relative mate size seemed to be important for female mate choice, and in the absence of courtship behavior, male size alone increased the spawning behavior of the female (Gaudemar et al., 2000). Yet, we also observed that females occasionally also spawned with smaller, sneaker males. A benefit to females in these cases may arise from exposing eggs to sperm from several males, resulting in higher genetic variation among offspring (Jennions & Petrie, 2000; Reichard, Le Comber, & Smith, 2007). Moreover, it is not unlikely that female charr may also incorporate a passive mate choice, yet actively choosing spawning ground and “nest” site. Under such a scenario, the outcome of the competition between the males in proximity to the selected “nest” site decides which male the female spawns with. In such case, mate guarding and social dominance among males become paramount. Thus, it seems like size‐dependent dominance among males including direct choice for male size might drive selection among males, but the two mechanisms may be hard to disentangle.

Salmonid males do not provide parental care, but larger males are better to chase away potential egg predators from the spawning site. Thus, females might derive direct benefits from spawning with large males through higher egg survival (Blanchfield and Ridgway, 1998; Berejikian, Tezak, & LaRae, 2000). Yet, in the present study, both the female and the guarding male were observed foraging on eggs from their own redd after spawning (unpublished data, Supporting information video S1). This observation of filial cannibalism is new for charr, and previous studies using similar approaches have not documented egg foraging among guarding males (Sigurjónsdóttir & Gunnarsson, 1989). Analysis of stomach contents has, however, shown that charr may eat eggs during the spawning period (Malmquist et al., 1992). Although intuitively maladaptive, eating own eggs is not uncommon among fish (review by Manica, 2002). Filial cannibalism has been explained as either removal of unfertilized, malformed, or diseased eggs, or by energy‐based arguments in species which have very high‐energy expenditures and limited foraging opportunities (Manica, 2002).

### 4.2 Synchrony

In sperm competition events, females experienced higher synchrony of gamete release with the guarding male than with the sneaker male(s). By releasing milt in high synchrony with the female, eggs pass through a cloud of milt in the water (Fitzpatrick & Liley, 2008), and when synchronizing the ejaculation with female egg release, the courting male may reduce the effect of sperm competition. In Atlantic salmon, a 2‐second delay in sperm release reduced paternity by approximately 40% in spawning events under sperm competition (Yeates et al., 2007). The average charr sneakers ejaculate their milt only 0.47 s after the guarding male, but the effect of sperm competition is necessarily not comparable in the two species. That is, unlike charr which spawn in still water, salmon spawn in flowing water, rendering the physical properties of the two fertilization environments quite different. Close imitations of natural sperm competition in charr show that when sneaker males release ejaculate 0.68 s after the guarding male, there is no difference in fertilization success (Egeland et al., 2015). That is, the initial higher sperm velocity and higher sperm numbers among sneakers may partly compensate for their lack of synchrony. Yet, this benefit might be outweighed by the sneakers’ lower sperm velocity in water‐diluted ovarian fluid compared to that of guarding males (Egeland et al., 2016). In single‐male spawning events, on the other hand, the sneaker males released their gametes with significantly higher synchrony than guarding males. The high synchrony exhibited by the sneakers when spawning singly suggests that sneakers’ lack of synchrony under sperm competition is caused by the mate guarding of the guarding male, rather than by the sneakers’ lack of ability to synchronize gamete release (Sørum et al., 2011). Thus, mate guarding seems to have a measurable effect on sneakers’ ability to synchronize their ejaculation with the egg release by the female.

### 4.3 Sperm competition

Although the female was guarded by one male in the lead‐up to every spawning situation, the guarding male could not prevent sperm competition. Approximately 50% of the observed spawning events occurred with sperm competition, and in these cases, around three males participated on average. Yet, compared to guarding males, sneakers experience a higher intensity of sperm competition, suggesting that there is an effect of guarding on the likelihood of experiencing sperm competition. Although females also show aggressive behavior toward sneaker males (unpublished data), females might have benefits from sperm competition. That is, eggs spawned under sperm competition are observed to achieve a higher fertilization success and a higher offspring survival relative to eggs fertilized by a single male (Keil & Sachser, 1998; Liljedal, Folstad, & Skarstein, 1999; Shapiro, Marconato, & Yoshikawa, 1994). Exposing eggs to sperm from several males may also result in higher genetic variation among offspring (Jennions & Petrie, 2000; Reichard et al., 2007). Yet, approximately 50% of the observed spawning events were single‐male spawning events. These events may have occurred either when the density of surrounding males was low or when the surrounding males were occupied in intrasexual interactions resulting in a late arrival to the spawning female. Thus, aggressive behavior from both the guarding male and the female may reduce the intensity of sperm competition, but the estimated number of interacting males in all spawning events (close to 2) hints to a situation where the ejaculate investments should be at the highest (Parker, Ball, Stockley, & Gage, 1996).

### 4.4 Male density

There was a clear increase in the number of males in proximity to the spawning female seconds before female egg release. Additionally, a similar increase is observed in “near” spawning events, where there is no release of neither male nor female gametes. This indicates that there is some other factor than gonadal products, or its associated chemical components, that are attracting males to the spawning couple. Signals within the spawning pair are thought to be perceived visually or by tactile sensation (Uematsu & Yamamori, 1982), but it is unlikely that the attractors for sneaker males are visual cues only. That is, individuals seen heading away from, and unable to see in the direction of the prespawning pair, are sometimes observed to rapidly turn and head for the spawning pair when the courtship quivering begins and before the actual spawning occurs (own observations). Additionally, the spawning individuals in a pair would also not be able to see gamete release from the partner (i.e., it occurs in a dead angle of his/her visionary field). Thus, communication signals related to spawning synchrony are most likely not visual, but rather vibrational. In captive experiments of spawning behavior of landlocked red salmon (*Oncorhynchus nerka*), visual patterns were not alone essential for eliciting the male spawning behavior. Yet, the vibrational and visual cues had to coincide spatially in order to elicit the male spawning behavior (Satou, Takeuchi, Takei, et al., 1994). From our videos, it seems like the spawning pair uses vibrational communication to synchronize the gamete release (Supporting information video S1) and this vibrational communication produces waves in the water column that can be recognized as sound (Figure 2, 3). This is, to our knowledge, the first time sound‐producing communication has been reported in Arctic charr and our finding is in contrast to Bolgan, O'Brien, Rountree, and Gammell (2016), who could not find evidence of acoustic signaling in Arctic charr during courtship. Thus, the observed prespawning increase in density could be caused by surrounding males picking up the vibrational signal used by the spawning pair, informing the sneakers about time and space of gamete release. This could explain the relatively short delay in sneakers’ milt release and the observed influx of males close to egg release. If vibrations attract males to the courting couple, it might be argued that a long vibrational period should attract more males than a shorter vibrational period. Yet, no correlation was found between vibrational period and the number of males present at the spawning event. Thus, rather than vibrational period, vibrational frequency might be the important component of the communication. This concurs with findings in landlocked red salmon where the male behavior was clearly influenced by the vibrational frequency of the model female (Satou, Takeuchi, Takei, et al., 1994). Similarly, male and female haddock (*Melanogrammus aeglefinus*) seem to synchronize reproductive behavior by sound from muscle vibrations as well (Hawkins & Amorim, 2000). Thus, the frequency of vibrations could be the main stimulus enabling the spawning pair to synchronize their gamete release. At the same time, the frequency might be the stimulus surrounding sneaker males use for eavesdropping to synchronize their spawning. Additionally, our study was conducted under daylight condition, and it should be noted that vibrational communication might be even more important at night when spawning commonly occurs under very restricted light conditions (own observations). Furthermore, this study was not specifically designed for sound recordings (see Introduction). The sounds were recorded by the built‐in microphone in the GoPro cameras enclosed within a watertight housing. Such microphones are made for recording airborne, and not waterborne, sounds. In future studies, we will record sounds from spawning charr using proper hydrophones. Such recordings have the potential to reveal more details about these sounds, such as frequency, amplitude, and length of the sounds recorded in different courtship and spawning events.

Throughout this study, mate guarding seems to be the prevailing factor for paternity in Arctic charr. Mate guarding affects accessibility to females, sperm competition, synchrony of gamete release, paternity, and subsequent egg predation. By obstructing competition, advantageous positioning, tailoring of sperm production, and synchronized milt release, a guarding male's sperm have increased chances of reaching the micropyle. Yet, a synchronized gamete release requires good communication, and charr seem to have developed signals to synchronize gamete release with the consequence of increased detectability by surrounding males, making vibrational communication a “double‐edged sword.”

## CONFLICT OF INTEREST

The authors have no conflict of interest to declare.

## AUTHOR CONTRIBUTIONS

Brattli MB, Egeland TB, Nordeide JT and Folstad I were involved in project development, design, analysis, and all contributed to the manuscript.

## DATA ACCESSIBILITY

Behavioral data are available at Dryad https://doi.org/10.5061/dryad.b8br852
